# Influence of Caloric Vestibular Stimulation on Body Experience in Healthy Humans

**DOI:** 10.3389/fnint.2016.00014

**Published:** 2016-03-11

**Authors:** Andreas Schönherr, Christian Albrecht May

**Affiliations:** Institute of Anatomy, Medical Faculty Carl Gustav Carus, Technical University DresdenDresden, Germany

**Keywords:** caloric vestibular stimulation, body experience, body schema, body awareness, body image

## Abstract

The vestibular system has more connections with and influence on higher cortical centers than previously thought. These interactions with higher cortical centers and the phenomena that they elicit require a structural intact cerebral cortex. To date, little is known about the role and influence of the vestibular system on one’s body experience. In this study we show that caloric vestibular stimulation (CVS) in healthy participants has an effect on the perceptive component of one’s body experience. After CVS all participants showed a statistically significant difference of thigh width estimation. In contrast to previous studies, which demonstrated an influence of CVS on higher cortical centers with an intact cerebral cortex both the cognitive and affective component of body experience were not effected by the CVS. Our results demonstrate the influence of the vestibular system on body perception and emphasize its role in modulating different perceptive-qualities which contributes to our body experience. We found that CVS has a limited influence on one’s conscious state, thought process and higher cortical functions.

## Introduction

The vestibular system, originally conceived as a reflex system to generate and control spinal and ocular-motor movements (Palla and Lenggenhager, 2014), appears more interesting today. During the last few years, an increasing number of articles concerning the vestibular system have demonstrated new innervations and influences than previously thought. It interacts with numerous higher cortical centers, which presume an intact cerebral cortex. These interactions include the interpretation of tactile and heat stimuli (Ferrè et al., 2013a), the spatial discrimination of tactile stimuli (Ferrè et al., 2013b), mental imagery (Péruch et al., 2011), attention (Figliozzi et al., 2005), memory (Smith et al., 2010), risk behavior (McKay et al., 2013), mood (Preuss et al., 2014a), social interaction (Deroualle and Lopez, 2014), and even the desire and probability of purchasing a product (Preuss et al., 2014b). However, little is known about the influence and role of the vestibular system on body experience; the present knowledge is primarily based on studies involving pathological conditions: people with vestibular diseases may show “out-of-body-experiences” (Skworzoff, 1931), perception of absent body motion (vertigo; Curthoys and Halmagyi, 1995) or symptoms of depersonalization and derealization (Smith and Darlington, 2013). Furthermore a distorted body experience, which is a central part of different diseases and situations, can be influenced by caloric vestibular stimulation (CVS). These situations and diseases include neglect and anosognosia (Ramachandran et al., 2004), somatoparaphrenia (Bisiach et al., 1991), pain (Mast et al., 2014), camptocormia (Okada et al., 2012), mania (Levine et al., 2012), shape and position of phantom-limbs (André et al., 2001), and also anorexia nervosa (Schönherr and May, 2015). The main question leading to the present study concerned the role of the vestibular system on body experience in healthy participants. Our study focuses on the influence of CVS on the four different components of one’s total body experience (perceptive, cognitive, affective and behavioral; Thompson, 1999). The perceptive component contains sensory information of all modalities regarding one’s own body. It includes the perception of the body’s dimensions as well as its orientation in space and is the basis of one’s body image. The affective component includes the feelings regarding one’s own body and is therefore the basis of one’s body awareness. The third component is the cognitive component regarding thoughts and attitude towards one’s own body. It is the basis of self image and ego. The last component is behavior and can be separated into motor (facial expressions, gestures, posture) and into social behavior. For a precise use of terms we adhered to the “Consensus Paper on terminological differentiation on various aspects of body experience” (Röhricht et al., 2005).

In the present study, we investigated the influence of CVS on different parts of body experience using established methods for perceptive (body-size estimation, silhouette-technique) and cognitive components (questionnaires) as described in the “Materials and Methods” Section. This work was part of a project which previously investigated the influence of caloric irrigation on body experience of anorectic patients in an attempt to open a new therapeutic window for their treatment (Schönherr and May, 2015). Surprisingly we could also identify a distinct influence of vestibular system stimulation in non-anorectic volunteers. The quality of this influence might help to explain why the stimulation technique did not show the same results in anorexia nervosa compared with neglect or other conditions listed above.

## Materials and Methods

Thirty students of the Medical Faculty Carl Gustav Carus in Dresden were recruited by flyers and advertisement in lectures. Based on their own statements they did not suffer from any psychological or physical diseases, and the exclusion criteria consisted of body mass index (BMI) under 17.5, amenorrhea, malposition or inflammation of the external auditory canal, perforated eardrum, diseases of the vestibular system (previously known or detected during clinical examination), and conditions known to change body experience that is e.g., pregnancy, anorexia nervosa, depression, panic-attacks, hypochondria.

To investigate the perceptive component of body experience, a body-size estimation technique (developed by Horn and Scholz, 2009) and a silhouette-technique (figure-scale developed by Thompson and Gray, 1995) were used. For body-size estimations, the students were asked to show the thigh width (in the middle of the thigh) with their hands (eyes covered). The thigh width was used since it has a reasonable size in order to detect small differences. On the basis of comparability the protocol of Horn and Scholz (2009) was not modified limiting the body-size estimation to one region of the lower extremity and to conditions without tactile stimuli. Using the silhouette-technique, the students had to identify their actual perceived figure, their actual felt figure and their ideal figure from seven figure drawings of different shapes. The cognitive-affective components of body experience were measured by questionnaires: the questionnaire for body image (FKB-20) presents the vital and dismissive body dynamic (Clement and Löwe, 1996) and the physical appearance state and trait anxiety score (PASTAS) measures body related fears (Reed et al., 1991). For stimulating the vestibular system we used a CVS with cold air, predominantly activating the horizontal semicircular canals (Palla and Lenggenhager, 2014). For practical reasons we chose the air-based CVS, even though we know about the converse discussion about comparability of water- and air-based methods (Zapala et al., 2008). The study was approved by the local ethic committee (EK53032007).

The examination contained two appointments (each between 5 and 8 pm) for separated stimulation of left and right vestibular organ with an interval of at least 24 h. After testing the requirements for CVS (ear mirroring using a portable otoscope, Unterberg-test, Romberg-test, nystagmus with Frenzel-glasses), the real thigh diameter (TW) was measured with an anthropometric calipers. After first estimation of TW (using both hands to show the felt TW with closed eyes), answering the questionnaires and a second estimation of TW before the CVS, the CVS was performed. Therefore we used the method according to Hallpike (1956) with 27°C tempered air (air-calorisator AIRMATIC II^™^ by Hortmann) for 45 s with a subsequent 1 min counting of caloric induced nystagmus (Frenzel-glasses) in a darkened room. The ear irrigation was followed by a third estimation of TW, another answering of the questionnaire and a fourth estimation of TW. After precisely adjusting the estimated TW with their hands (and covered eyes) before and after the CVS, the patients were invited to open their eyes and observe their estimated TW. The reaction of the participants regarding differences between estimated and real TW was not evaluated in this study. We used the Body-Perception-Index (Slade and Russell, 1973) as a quotient of estimated and real measurement of TW, which we calculated in the following way:

$$BPI(\%)\;=\;\left(\frac{estimatedamount}{realamount}\right)*100$$

A BPI over 100% means an overestimation and a BPI under 100% underestimation of the real body dimension.

For statistical analysis we used the Software SPSS (IBM) and the Wilcoxon signed-rank test for related samples. We compared the effect of CVS (induced eye movements) between left and right side, the results of body-size estimation before and after CVS respectively for left and right side and the change of BPI after CVS of right and left side.

## Results

### Characteristics of the Cohort

The sample (*n* = 30) contained 22 females and 8 males participants (P) between 18 and 31 years (φ 24.6 ± 2.9 years). Body size was located between 1.55 and 1.97 m (φ 1.73 ± 0.97 m) and body weight between 51 and 100 kg (φ 67 ± 13 kg). The BMI as an indicator of the size-related weight was between 18.9 and 26.4 kg/m^2^ (φ 22.3 ± 2.2 kg/m^2^). Twenty-eight participants were right-handed and two left-handed. One participant reported recurrent vertigo attacks in stressful situations, two about hypothyroidism with intake of L-thyroxin, and two others about a nodular goiter. Eighteen of 22 females participants were on oral contraception. Two participants (P11/P16) reported recurrent distance-problems, which means, that they bump into things very often. A slimmer body was favored by 23 of 30 participants, six participants showed a desired body figure which corresponds to their actual perceived body figure, and one participant wanted a plumper figure (Additional aspects can be found in attachment 1).

### Estimation of Thigh Width

**Figure 1:**
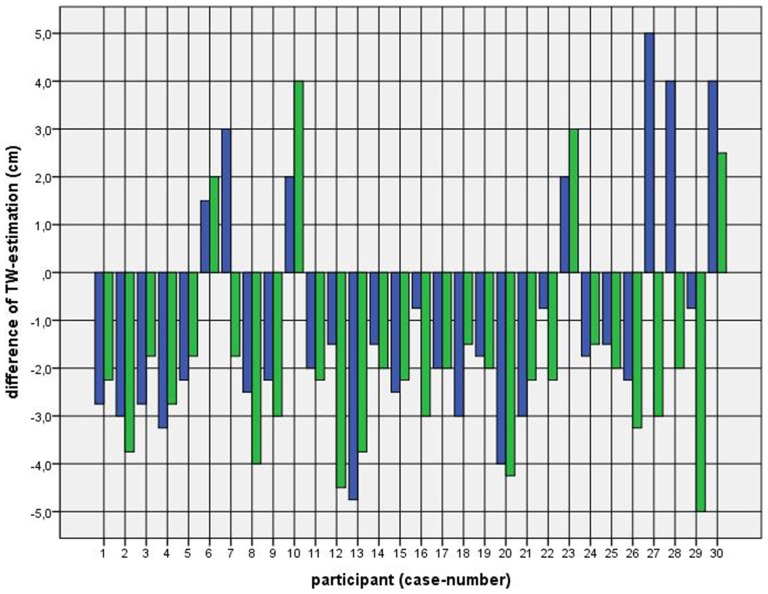
**Difference between mean of thigh width (TW) estimation before and first TW estimation after caloric vestibular stimulation (CVS).** *Key*: blue—left-CVS; green—right-CVS.* Annotation*: negative difference—first estimated TW after CVS was thinner than mean of TW-estimation before CVS; positive difference—first estimated TW after CVS was wider than mean of TW-estimation before CVS.

**Figure 2:**
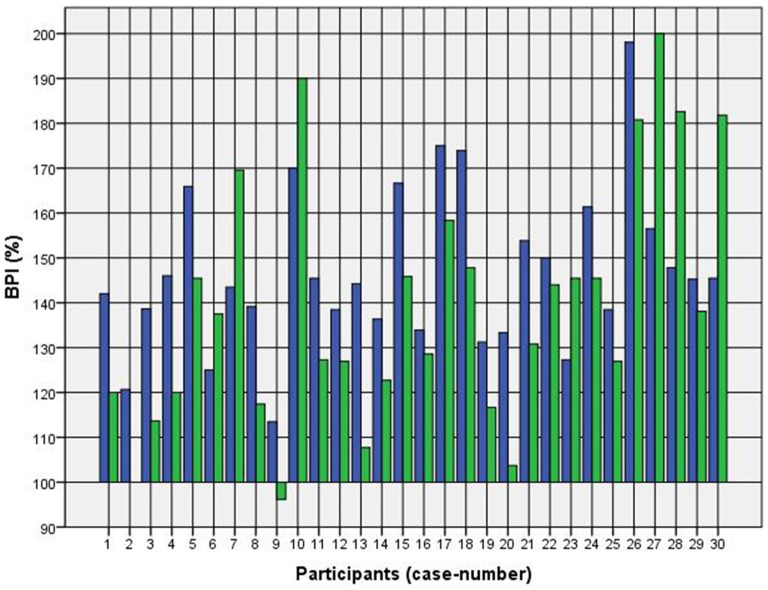
**Body Perception Index (BPI) before and after left CVS.** *Key*: blue—BPI before CVS; green—BPI after CVS*. Annotation*: BPI > 100% means overestimation of TW; BPI < 100% means underestimation of TW.

**Figure 3:**
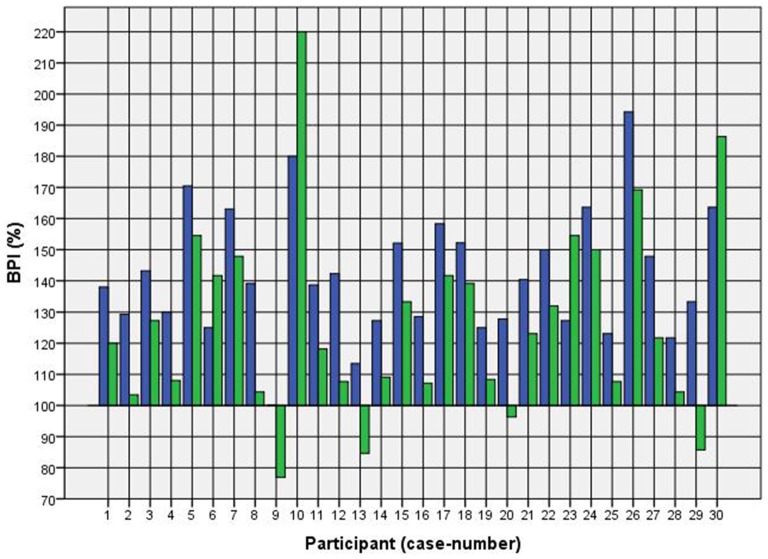
**BPI before and after right CVS.** *Key*: blue—BPI before CVS; green—BPI after CVS*. Annotation*: BPI > 100% means overestimation of TW; BPI < 100% means underestimation of TW.

**Figure 4:**
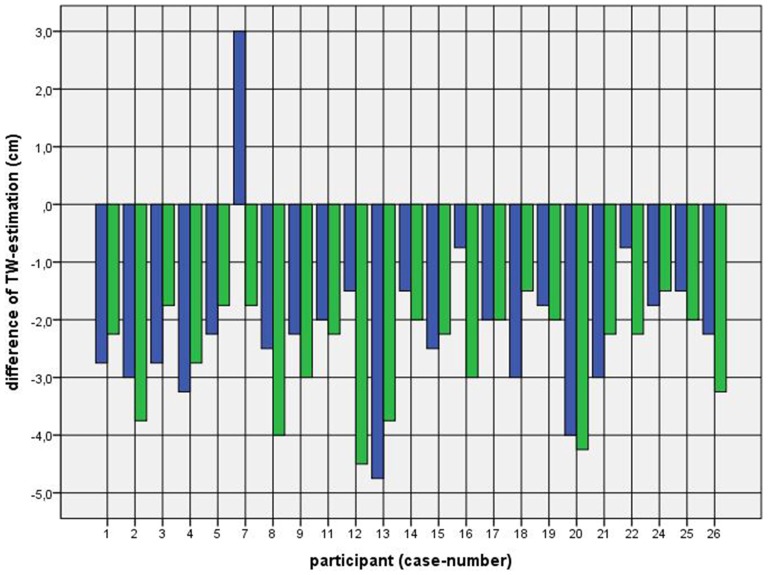
**Difference between mean of TW estimation before and first TW estimation after CVS among participants with a favored figure thinner than their actual perceived.** *Key*: blue—first appointment, left-CVS; green—second appointment, right-CVS. *Annotation*: negative difference—first estimated TW after CVS was thinner than mean of TW-estimation before CVS; positive difference—first estimated TW after CVS was wider than mean of TW-estimation before CVS.

**Figure 5:**
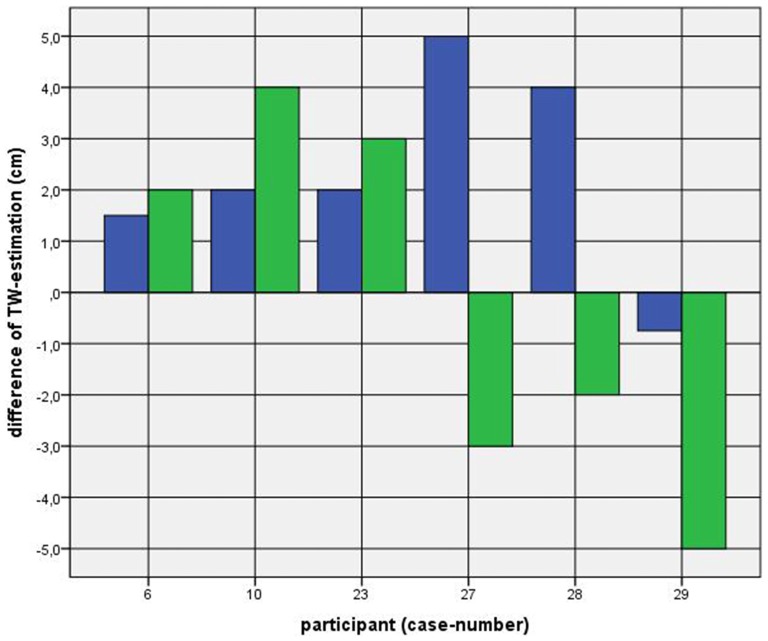
**Difference between mean of TW estimation before and first TW estimation after CVS among participants with a corresponding favored and actual figure.** *Key*: blue—first appointment, left-CVS; green—second appointment, right-CVS.* Annotation*: negative difference—first estimated TW after CVS was thinner than mean of TW-estimation before CVS; positive difference—first estimated TW after CVS was wider than mean of TW-estimation before CVS.

Because of the small difference of 0.5 cm between the first and second basal TW estimation we selected the mean and compared it with the first estimation after CVS. The difference before-after was 0.8–5.0 cm (φ 2.5 ± 1.1 cm) on the left and 1.5–5.0 cm (φ 2.7 ± 1.0 cm) on the right side (Figure 1). This was statistically significant in the 5% level (Wilcoxon signed-rank test for related samples). The difference before-after was greater or equal 1.5 cm in 27 participants. Most participants (*n* = 23) estimated their TW thinner than before, four participants estimated their TW wider, and three participants showed a difference between left and right side (Figure 1). The change in TW-estimation can also be seen in the Body Perception Index (BPI; Figures 2, 3): before CVS a general overestimation (BPI > 100%) was present with mild differences between the right and left side. Comparing the TW-estimation with the desired body-figure, we observed that all participants who favored a slimmer desired figure (except P7 after left CVS) estimated their TW after CVS of both sides thinner than before (Figure 4). This was not statistically significant. It is only an observed phenomenon. The participants, whose desired and actual figure matched, showed no clear direction in the change of TW-estimation CVS (Figure 5). The one participant who favored to be plumper estimated his TW wider after CVS. Even though a measurable change in TW-estimation after CVS was present in every participant, 26 of them were not aware of it. They were convinced of showing the same TW every time. Only four participants were uncertain with their TW-estimation after CVS, but did also not become aware of the present change.

Oral contraception had no specific effects on the tested outcome.

### Questionnaires

The figure scale, the FKB-20 and the PASTAS showed no basal peculiarities and no statistically significant changes after CVS (A detailed evaluation can be found in attachment 2).

## Discussion

### Influence on Perceptive Component

All participants showed a statistically significant change in their TW estimation after CVS. This might indicate that the vestibular system has a decisive role in the body image and the perceptive component of one’s body experience. CVS seems to influence the components for perceiving the physical limits of one’s body. This confirms the previous work (Ferrè et al., 2013a) concerning the influence of the vestibular system on the body-image. However, the method of CVS on body image related measurements was not tested by non-body-related spatial distances. Therefore, a possibility of non-specific reaction on CVS remains to be ruled out and has to be addressed in further studies. In addition, the measurements included only the thigh which changes its width considerably between standing and sitting. It remains to be determined if estimations in other regions of the body show similar changes after CVS and if the thigh is therefore representative for this kind of perceptive body experience. Furthermore, no tactile stimuli for body size perception were included in this study. The estimation might therefore partially depend on cognitive aspects.

Participants, who wanted to be thinner, estimated their TW smaller after a CVS. Their TW overestimation was reduced after CVS. According to the theory of “sensory signal management” (Bottini et al., 2013) a vestibular stimulation can modulate sensory input between the environment and the body. This modulation can help to adjust information and better predict forthcoming perceptions with their resulting actions (Mast et al., 2014). Thus, it is conceivable that a vestibular stimulation might modulate the internal picture of the thigh in favor of a desired body image. The desired image might influence the somatosensory perceptual qualities, tonus and postural control which enable the adjustment of an “unaware, reality based, desired image”.

Furthermore, the majority of participants did not become consciously aware of this change. We suppose that CVS is able to influence higher cortical centers but does not reach the level of consciousness. This is supported by the lack of changes within the questionnaires which require an awareness and conscious state. Although there is a known influence on the perceptive component of body experience, it‘s detection with the figure scale shows no statistically significant change after CVS. We conclude that a vestibular stimulation as performed in this study is not intense enough to reach the level of one’s conscious state.

### Influence on Cognitive-Affective Component

The specific cognitive-affective component, studied with questionnaires, revealed no statistically significant changes after CVS. However, some evidence from literature support the influence of the vestibular system on different phenomena requiring structural integrity of the cortex. Current studies show an influence of CVS on one’s body-integrity, awareness and image (Ferrè et al., 2014) and on body movement (Guillaud et al., 2011). Why were we not able to demonstrate the influence of CVS on cognitive-affective components in our study? It might be possible that the intensity of caloric irrigation was not strong enough to reach the higher cortical centers. According to this we would recommend a stronger irrigation method (e.g., cold water) in future tests.

However, another aspect might also be taken into account: the vestibular system is a phylogenetic old system, located in the brain stem and inner ear. Although there are multiple synapsis in the cortex (Lopez et al., 2012), which can lead to a cortical processing and an influence of vestibular information, the vestibular system might not reach all areas in the phylogenetically younger cortex, necessary for one’s conscious experience and reflection. We think that the questionnaires are probably not suitable for studying the influence of the vestibular system on body experience. Activities like the TW-estimation appear to better demonstrate the distorting influence of the caloric vestibular system on body experience.

A third aspect could also account for the steady answers of the questionnaires: cognitive body experiences might reflect a more steady state, and therefore less likely to be influenced by situational factors like CVS.

## Author Contributions

All authors listed, have made substantial, direct and intellectual contribution to the work, and approved it for publication.

## Conflict of Interest Statement

The authors declare that the research was conducted in the absence of any commercial or financial relationships that could be construed as a potential conflict of interest.
