# Supplementary material for: Influence of Caloric Vestibular Stimulation on Body Experience in Healthy Humans
Source: Front Integr Neurosci. 2016 Mar 11;10:14. doi: 10.3389/fnint.2016.00014 (PMC4787111; doi:10.3389/fnint.2016.00014)
Supplement: Supplementary file 1 [file DataSheet_1.docx]

**Additional information:**

**Attachment 1. Results concerning the state and reactivity of the vestibular system:**

In all subjects, the Romberg-test was negative. The Unterberg-test showed some abnormalities: two participants (P16/P28) showed a reproducible deviation over 45° (and < 60°) to the left, four participants (P17/P18/P21/P22) to the right site with a side-corresponding exhaustible nystagmus in calm.

After caloric irrigation we counted between 11 and 70 (φ 38 ± 19) caloric-induced eye movements on the right and between 7 and 69 (φ 33 ± 18) on the left side. Both vestibular organs showed considerable differences in their reaction to stimulation (Figure A1). Four participants complained about nausea after CVS of both sides, one after CVS of right and one after CVS of left side. 20 participants reported about vertigo after CVS in both sides, four after right-side CVS and three after left-side CVS.

**Figure A1 - Difference of one-minute-counted caloric-induced nystagmus between left- and right-side caloric irrigation.** *Annotation:* a positive difference means, the caloric-induced nystagmus was left higher than right and a negative difference means, the caloric-induced nystagmus was right higher than left.


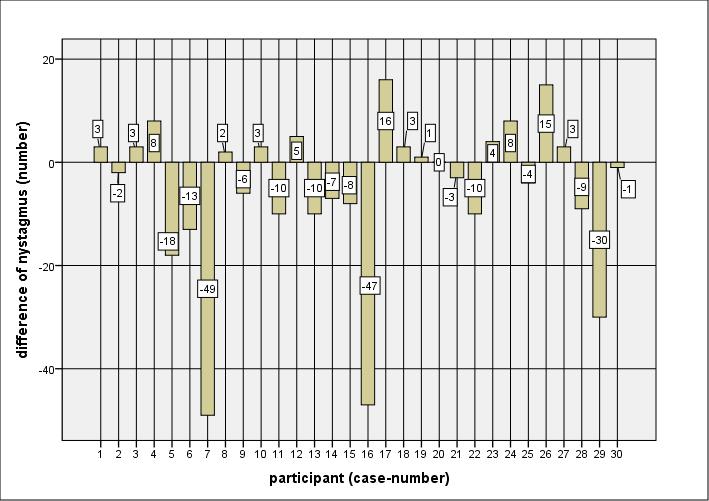


**Attachment 2. Detailed evaluation of the questionnaires**

In the figure scale the actual felt figure shows a difference between -1 and 1 (φ 0 ± 1) scale units after CVS on the right side and between -1 and 1 (φ 0 ± 1) scale units after CVS on the left side and were not statistically significant in the Wilcoxon Test (left side CVS: p = 0,564 and right side CVS: p = 0,564). The actual perceived figure shows a difference between -1 and 1 (φ 0 ± 1) scale units after CVS on the right side and between -1 and 1 (φ 0 ± 1) scale units after CVS on the left side and were not statistically significant in the Wilcoxon Test (left side CVS: p = 1 and right side CVS: p = 1). The desired figure shows a difference between -1 and 1 (φ 0 ± 1) scale units after CVS on the right side and between -1 and 1 (φ 0 ± 1) scale units after CVS on the left side and were not statistically significant in the Wilcoxon Test (left side CVS: p = 1 and right side CVS: p = 1). So the figure-scale showed no statistically significant change in the desired, actual perceived and felt figure after CVS on both sides.

In the questionnaire for body image (FKB-20) the vital body dynamic scale shows a difference between 0 and 10 (φ 2 ± 3) scale units after CVS on the right side and between 0 and 11 (φ 2 ± 2) scale units after CVS on the left side and were not statistically significant in the Wilcoxon Test (left side CVS: p = 0,334 and right side CVS: p = 0,668). The dismissive body dynamic scale shows a difference between 0 and 6 (φ 2 ± 2) scale units after CVS on the right side and between 0 and 7 (φ 2 ± 2) scale units after CVS on the left side and were also not statistically significant in the Wilcoxon Test (left side CVS: p = 0,245 and right side CVS: p = 0,935). In the physical appearance state and trait anxiety score (PASTAS) the scale of fears regarding body parts, which are closely connected to body weight (“state” scale) shows a difference between -4 and 2 (φ 0 ± 2) scale units after CVS on the right side and between -6 and 3 (φ 0 ± 2) scale units after CVS on the left side and were not statistically significant in the Wilcoxon Test (left side CVS: p = 0,171 and right side CVS: p = 0,476). The scale of fears regarding body parts, which are not connected to body weight (“trait” scale) shows a difference between -7 and 1 (φ 0 ± 2) scale units after CVS on the right side and between -4 and 3 (φ 0 ± 1) scale units after CVS on the left side and were also not statistically significant in the Wilcoxon Test (left side CVS: p = 0,85 and right side CVS: p = 0,299). So both questionnaires showed no statistically significant difference of self-assessment of the body experience after CVS on both sides.
